# Wavelet Packet Singular Entropy-Based Method for Damage Identification in Curved Continuous Girder Bridges under Seismic Excitations

**DOI:** 10.3390/s19194272

**Published:** 2019-10-02

**Authors:** Dayang Li, Maosen Cao, Tongfa Deng, Shixiang Zhang

**Affiliations:** 1 Department of Engineering Mechanics, Hohai University, Nanjing 210098, China; 2 Jiangxi Provincial Key Laboratory of Environmental Geotechnical Engineering and Disaster Control, Jiangxi University of Science and Technology, Ganzhou 341000, China; 3 China Design Group Co., Ltd., Nanjing 210014, China

**Keywords:** wavelet packet singular entropy, structural health monitoring, seismic damage, damage identification, dynamic response, curved continuous girder bridge, seismic excitation

## Abstract

Curved continuous girder bridges (CCGBs) have been widely adopted in the civil engineering field in recent decades for complex interchanges and city viaducts. Unfortunately, compared to straight bridges, this type of bridge with horizontal curvature is relatively vulnerable to earthquakes characterized by large energy and short duration. Seismic damage can degrade the performance of CCGBs, threatening their normal operation and even resulting in collapse. Detection of seismic damage in CCGBs is thus significantly important but is still not well resolved. To this end, a new method based on wavelet packet singular entropy (WPSE) is proposed to identify seismic damage by analyzing the dynamic responses of CCGBs to seismic excitation. This WPSE-based approach features characterizing damage using synergistic advantage of the wavelet packet transform, singular value decomposition, and information entropy. To testify the algorithm, a finite element model of a typical CCGB with two types of seismic damage is built, in which the seismic damage is individually modeled by stiffness reductions at the bottom of piers and at pier-girder connections. The displacement responses of the model to El Centro seismic excitation is used to identify the damage. The results show that damage indices in the WPSE-based approach can correctly locate the seismic damage in CCGBs. Furthermore, the WPSE-based method is competent to identify damage with higher accuracy in comparison with the wavelet packet energy based method, and has a strong immunity to noise revealed by robustness analysis. An array of responses used in this approach paves the way of developing practical technologies for detecting seismic damage using advanced distributed sensing techniques, typically the optical sensors.

## 1. Introduction

Curved continuous girder bridges (CCGBs) are key components of urban traffic, extensively used as complex interchanges and city viaducts in civil infrastructure [1,2], due to their improved structural performance including strong ductility and energy consumption capability under earthquakes and strong dynamic excitations. To ensure their safety in service, a number of analytical and experimental studies of the complex static and dynamic behavior of CCGBs [3,4,5,6] have been performed. In particular, Rodgers et al. [7] reported that the structural responses of CCGBs are more complex than expected, due to their highly symmetrical geometry with significant torsion during strong shaking at the top of concrete piers.

In recent years, the health assessment of CCGBs after earthquake has attracted much attention in the interest of ensuring safe operation. The visual inspection for local and visible flaw [8,9,10] is an extensively adopted method for condition assessment of CCGBs after earthquake; nevertheless, such a method works with strongly dependent on subjective judgment and lacking the capability of evaluating the strength and/or deformation capacity reserve of a bridge. In recent decades, structural health monitoring (SHM) has become an innovative way of inspecting structural damage in a rapid, remote, automated, and objective fashion, especially with the rapid development of advanced sensing technologies, such as distributed optical fiber sensors [11,12]. However, there is as yet a lack of advanced damage identification methods matched with such advanced sensing technologies. Existing SHM based damage detection methods employ dynamic characteristics such as natural frequencies [13,14,15,16,17], mode shapes [18,19,20,21], modal curvatures [22,23,24,25], and wavelet transform coefficients [26,27,28], to establish damage features. Among those methods, wavelet transform based diagnosis of damage has been adopted extensively with advantages in the time-frequency and multi-resolution analysis of measured dynamic responses of structures.

Representative studies of wavelet transform based damage identification of bridge structures subjected to seismic excitations are described as follows. Wang and Chan [29] reported that wavelet coefficients showed more sensitivity than the original signals to local changes in structural properties. Cruz and Salgado [30] compared six damage detection methods based on vibration monitoring in two case studies of bridges. They concluded that wavelet transform based methods could identify damage location successfully, with the wavelet packet signature producing the best performance for noisy data and non-extensive damage. Todorovska and Trifunac [31] utilized the highest resolution detailed sub-band from multiresolution analysis with expansion on the basis of bi-orthogonal wavelets to detect novelties in recorded seismic responses. They concluded that the method could identify the time of occurrence and roughly the spatial distribution and degree of the major damage in the imperial county services (ICS) building. Vafaei and Adnan [32] investigated the applicability of the continuous wavelet transform (CWT) and discrete wavelet transform (DWT) to seismic damage detection of tall airport traffic control towers, numerically proving that CWT could successfully detect seismic damage even with noise-polluted signals. Aguirre et al. [33] explored the feasibility of using output-only model-free wavelet-based techniques for damage detection in reinforced concrete structures subjected to seismic excitations. They numerically and experimentally revealed that wavelet analysis methods were capable of identifying rebar fracture episodes and partially detecting frequency shifts in structures as the inelastic demand increased. Bagheri and Kourehli [34] proposed a DWT-based damage diagnosis method for structures under seismic excitation, with the effectiveness numerically demonstrated by analysis of a concrete shear wall and the first phase of an IASC-ASCE benchmark structure. Balafas and Kiremidjian [35] studied several data-driven damage sensitive features based on the CWT of both input acceleration signal and output acceleration response. Kaloop et al. [36] applied wavelet analysis methods when investigating the performance of an administration building during earthquake shaking. They indicated that the wavelet spectrum could illustrate the dominant frequency and reveal the elasticity responses of the structure during such shaking. Furthermore, Kaloop and Hu [37] adopted the energy of wavelet transform and correlation coefficients to detect the performance of a damaged building, with the feasibility numerically verified by regular and irregular simulation models manifesting that the energy of DWT showed significantly superior performance to that of CWT in detecting damage to the building.

Apart from wavelet transform coefficients, various methods based on wavelet entropy, wavelet packet energy entropy, and wavelet singular entropy have been developed to identify damage in bridges in recent years. Ren and Sun [38] provided wavelet entropy based features by combining the wavelet transform and the Shannon entropy, including wavelet entropy, relative wavelet entropy, and wavelet-time entropy. Their numerical and experimental cases showed that wavelet entropy based methods were effective to detect and locate structural damage. Diao et al. [39] proposed an entropy based two-step method for identifying damage in an offshore platform under seismic excitation. They argued from numerical and experimental cases that the entropy based two-step methodology could provide satisfactory damage identification results. Lee et al. [40] proposed a continuous relative wavelet entropy based reference-free damage detection algorithm for truss bridge structures. They emphasized that the method was sensitive to slight damage and suitable for highly nonlinear and nonstationary random response data due to the multiresolution signal analysis feature of CWT. He et al. [41] presented a wavelet packet energy entropy based method for structural damage identification using impulse responses. They experimentally revealed that the proposed wavelet packet energy entropy based method was capable of identifying single and multiple damage in pile structures. Li et al. [42] proposed a wavelet singular spectrum entropy to evaluate the damage condition of building structures, with the applicability of the proposed method numerically and experimentally verified by the damage analysis of a timber structure. In summary, the entropy based method has advantages in improving the accuracy of damage identification and shows significant applicability to damage detection in bridges, especially in curved bridges.

Most extant methods have been focused on detecting damage in straight bridges subjected to seismic excitation, with emphasis on revealing abnormality of wavelet coefficients of dynamic responses to characterize damage. However, CCGBs are more complex than straight bridges, because the horizontal curvature makes curved bridges more vulnerable to extensive damage and possible collapse. There are few dedicated methods for detecting seismic damage for CCGBs, especially fairly lacking effective methods that match the advanced distributed optical measurement.

To this end, this study develops a method for health assessment and damage identification in CCGBs. A new wavelet packet entropy based method is proposed to identify seismic damage by analyzing the dynamic responses of CCGBs subject to seismic excitation. This method is employed to identify two types of seismic damage in CCGBs, located at the bottom of piers and at pier-girder connections, respectively. Damage identification in numerical CCGB model subjected to El Centro seismic excitation shows the superiority of the proposed method in damage identification accuracy and noise immunity. The proposed method is expected to provide an effective and applicable tool for damage identification using dynamic responses recorded by advanced sensing techniques, especially by distributed optical fiber sensors.

The rest of the paper is organized as follows. The fundamentals of wavelet packet, singular value decomposition, and information theory are introduced in Section 2. A wavelet packet entropy based algorithm for damage identification is presented in Section 3. The damage model of the CCGB is fabricated in Section 4. Identification of damage in the CCGB is presented in Section 5, and discussion of influential factors on algorithm effectiveness is presented in Section 6.

## 2. Fundamentals

This section introduces the fundamentals of the wavelet packet transform (WPT), singular value decomposition (SVD), and information entropy (IE).

### 2.1. Wavelet Packet Transform (WPT)

The WPT is defined by the following recursive relationships [43]:

(1) $$\left\{\begin{array}{cc} u_{2n}^{\left(j\right)}\left(t\right) & =\sqrt{2}\sum_{k}h\left(k\right)u_{n}^{\left(j\right)}\left(2t-k\right)\\ u_{2n+1}^{\left(j\right)}\left(t\right) & =\sqrt{2}\sum_{k}g\left(k\right)u_{n}^{\left(j\right)}\left(2t-k\right) \end{array};n,k=0,1,2,\cdots ,\right.$$

where *j*, *k*, and *n* are the decomposition level, translation factor, and modulation parameter, respectively. The terms h(k) and g(k), satisfying $g\left(k\right)={\left(-1\right)}^{k}h\left(1-k\right)$, are quadrature mirror filters associated with the scaling function and the mother wavelet function. The scaling function is $u_{0}^{\left(0\right)}\left(t\right)=\phi \left(t\right)$, and the mother wavelet function is $u_{1}^{\left(0\right)}\left(t\right)=\psi \left(t\right)$.

The WPT contains complete decomposition at each level to achieve a higher resolution in the high frequency region at the next level. The recursive processes between the $j^{th}$ and the ${\left(j+1\right)}^{th}$ level of WPT are:

(2) $$\left\{\begin{array}{c} f_{j}^{i}\left(t\right)=f_{j+1}^{2i-1}\left(t\right)+f_{j+1}^{2i}\left(t\right)\\ f_{j+1}^{2i-1}\left(t\right)=Hf_{j}^{i}\left(t\right)\;\;,\\ f_{j+1}^{2i}\left(t\right)=Gf_{j}^{i}\left(t\right) \end{array}\right.$$

where *H* and *G* are filtering-decimation operators, that represent the low-pass filter and the high-pass filter, respectively. They can be obtained from the discrete filters h(k) and g(k) through:

(3) $$\left\{\begin{array}{cc} H\left\{\cdot \right\} & =\sum_{k=-\infty }^{\infty }h\left(k-2t\right)\\ G\left\{\cdot \right\} & =\sum_{k=-\infty }^{\infty }g\left(k-2t\right) \end{array}\right..$$

After being decomposed *j* times, the sum of WPT sub-bands can represent the original signal f(t) as

(4) $$f\left(t\right)=\sum_{i=1}^{2^{j}}f_{j}^{i}\left(t\right).$$

The WPT sub-band $f_{j}^{i}\left(t\right)$ can be derived by the linear superposition of wavelet packet functions $\psi_{j,k}^{i}\left(t\right)$ as

(5) $$f_{j,k}^{i}\left(t\right)=\sum_{k=-\infty }^{\infty }c_{j,k}^{i}\psi_{j,k}^{i}\left(t\right),$$

where $c_{j,k}^{i}$ is the coefficient of sub-band *i* at decomposition level *j* and can be calculated from:

(6) $$c_{j,k}^{i}=\int_{-\infty }^{\infty }f\left(t\right)\psi_{j,k}^{i}\left(t\right)dt.$$

All coefficients at the $j^{th}$ level construct a matrix C, containing the hidden intrinsic information in both low and high frequency regions, especially the higher resolution in high frequency regions.

### 2.2. Singular Value Decomposition (SVD)

According to the theorem of the SVD, a matrix A with the size m×n can be decomposed as

(7) $$A_{m\times n}=U_{m\times m}\Lambda_{m\times n}V_{n\times n}^{T},$$

where U and V are unitary matrices and their columns are orthonormal bases satisfying ${\mathrm{UU}}^{T}=I$ and ${\mathrm{VV}}^{T}=I$; Λ is a diagonal matrix with non-negative diagonal elements $\lambda_{i}$ sorted in descending order, that is, $\lambda_{1}\geq \lambda_{2}\geq \cdots \geq \lambda_{l}\geq 0$. These diagonal elements are called the singular values of the matrix A.

By selecting the dominant singular values, a large matrix can be represented by a smaller one without losing its major characteristic. This feature provides the theoretical basis for the broad applications of SVD [44,45,46,47].

### 2.3. Information Entropy (IE)

Information entropy is a measure of uncertainty. Suppose $p_{i}$ is the probability of output *i* and *N* is the total number of all probable outputs, then the information entropy of this type of source can be defined by the following Shannon’s formulation:

(8) $$H=-\sum_{i=1}^{N}p_{i}log_{10}p_{i}.$$

Shannon’s entropy defined by Equation (8) is efficient in quantifying the uncertainty and complexity of a source. The higher the *H*, the more complex is the system. It has been adopted in recent decades as a feature extraction tool in SHM [48,49].

## 3. Algorithm for Damage Identification

### 3.1. Damage Indices

For a measured vibration signal, $x=\left\{x(t)\right\}{|}_{t=1,2,\cdots ,N}$, together with the application of WPT in Equation (6), a coefficient matrix at the $j^{th}$ decomposition level, $D_{m\times n}^{j}$, can then be obtained as

(9) $$D_{m\times n}^{j}=\left[c_{j}^{1},c_{j}^{2},\cdots ,c_{j}^{n}\right],$$

where $c_{j}^{i}$ is the coefficient vector of the $i^{th}$ sub-band at the $j^{th}$ decomposition level; *m* is the row number representing the length of wavelet packet sub-bands; $n=2^{j}$ is the column number representing the total number of wavelet packet sub-bands. For convenience, $D_{m\times n}^{j}$ is hereinafter denoted as D.

Singular value decomposition can be applied to a coefficient matrix according to Equation (7):

(10) $$D=U\Lambda V^{T}.$$

Then, following Equation (8), the wavelet packet singular entropy of signal x is defined as

(11) $$WPSE=-\sum_{i=1}^{q}p_{i}log_{10}p_{i},$$

where

(12) $$p_{i}=\frac{\lambda_{i}}{\sum_{i=1}^{q}\lambda_{l}};$$

$\lambda_{i}$ and $\lambda_{l}$ are the diagonal elements of matrix Λ; *q* is number of selected singular value orders.

Wavelet packet singular entropy takes synergistic advantage of WPT, SVD, and IE, with the characteristics manifested in the following aspects: (1) WPSE retains the high time frequency multiresolution characteristics of WPT, permitting highlighting and characterization the intrinsic peculiarity of damage in the full frequency band; (2) WPSE retains the feature space mapping characteristics of SVD, enabling quantificational extraction the linear independent features from the wavelet space; (3) WPSE retains the system complexity metric characteristics of IE, facilitating characterization of the information feature. In summary, WPSE can highlight, extract, and quantificationally characterize the information characteristics, allowing it to be used as a characteristic factor for damage identification.

A WPSE-based damage index is defined as

(13) $$DI_{WPSE}=\frac{|WPSE^{d}-WPSE^{h}|}{WPSE^{h}},$$

where superscripts “${}^{h}$” and “${}^{d}$” denote healthy and damaged status, respectively. $DI_{WPSE}$ represents the relative difference ratio between the two statuses, quantificationally formulating the occurrence of damage. Because WPSE is positive according to Equations (11) and (12), the value of $DI_{WPSE}$ tends to be zero if the structure is in a healthy status. If the structure is in a damaged status, the value of $DI_{WPSE}$ at a damage location increases to be a positive number of greater magnitude than those in its neighborhood. Thus, $DI_{WPSE}$ can be used to identify and locate structural damage.

To demonstrate the damage location more clearly, a threshold value is established with the application of the one-sided confidence limit of $DI_{WPSE}$ from successive measurements [38]:

(14) $$DI_{WPSE}^{TH}=\mu +Z_{\alpha }\left(\frac{\sigma }{\sqrt{n}}\right),$$

where *n* is the total number of sensors distributed in a structure; μ and σ are the mean value and the standard deviation of the *n* damage indices; $Z_{\alpha }$ is the value of a standard normal distribution such that the cumulative probability is 100(1−α)%, and α represents the significance level. $DI_{WPSE}^{TH}$ can be considered as a threshold value that is an entrance point to possible abnormality in the damage feature. From a statistical point of view, the location of sensors whose $DI_{WPSE}$ values exceed the $DI_{WPSE}^{TH}$ implies an area where possible damage exists.

Further, the damage pre-warning index (DPWI) is defined as

(15) $$DPWI=DI_{WPSE}-DI_{WPSE}^{TH}.$$

The index DPWI specifies the differences between damage indices $DI_{WPSE}$ and their thresholds $DI_{WPSE}^{TH}$. Thus, the sign of DPWI indicates the structural status: a negative sign implies that the structure is healthy and there is no damage, whereas a positive sign indicates that the structure is unhealthy at the corresponding measured point where the damage is located. This kind of damage criterion is convenient for use in practical engineering. It is still recommended, however, to pre-set a positive DPWI as a warning value to reduce false alarms caused by measurement error, environmental noise, or other interferences.

### 3.2. Procedure of Damage Identification

The basic procedures of the WPSE-based methodology for damage identification of CCGBs subjected to seismic excitation are as follows:

Step 1: Measure the dynamic responses of the investigated CCGB subject to an earthquake excitation, along with consideration of the least favorable input angle and specific sensor arrangement.
Step 2: Calculate the value of damage index DPWI after completion of the following preparatory work:
  (a) determine the appropriate effective structural dynamic responses from measuring various types of responses in different directions;
  (b) select the optimal wavelet parameters used in WPT, including wavelet basic function and decomposition level;
  (c) choose the dominant order of singular values in calculating WPSE to eliminate the influence of noise.
Step 3: Identify damage in CCGBs with the constructed warning curve according to DPWIs.

**Figure 1:**
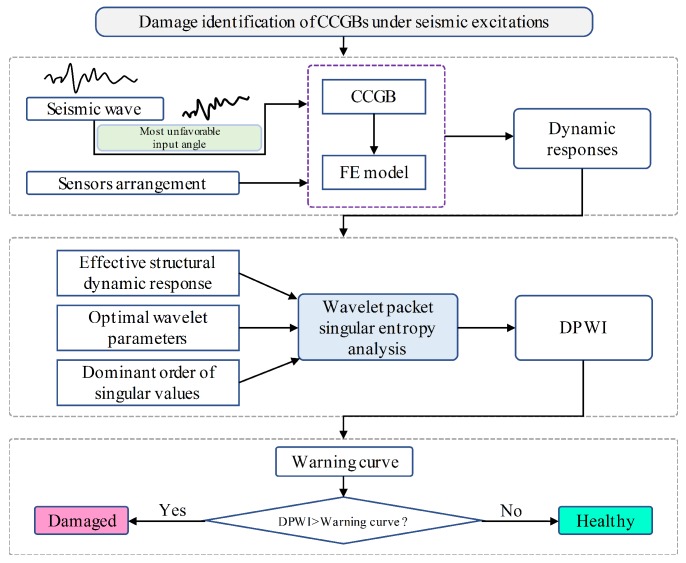
Roadmap of WPSE-based damage identification method for CCGBs.

These procedures are illustrated in Figure 1. Verification and detailed discussion of the proposed methodology are presented in the following sections.

## 4. Damage Model of CCGB

The damage model of a real investigated CCGB is elaborated in this section. Two typical damage types are considered, with damage individually located at the bottom of piers and at pier-girder connections. Sixteen damage scenarios are configured. The El Centro seismic excitation is applied to generate the displacement dynamic responses, which are measured by sensors arranged on the curved bridge.

### 4.1. Finite Element Model

**Figure 2:**
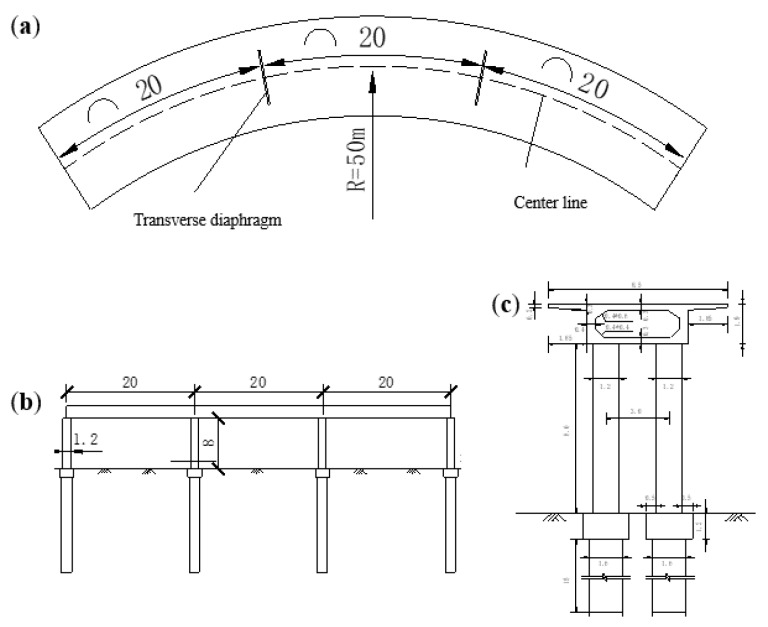
Geometry of the CCGB: (**a**) plane view; (**b**) elevation view; (**c**) cross-sections of piers and curved girder; unit: m.

**Figure 3:**
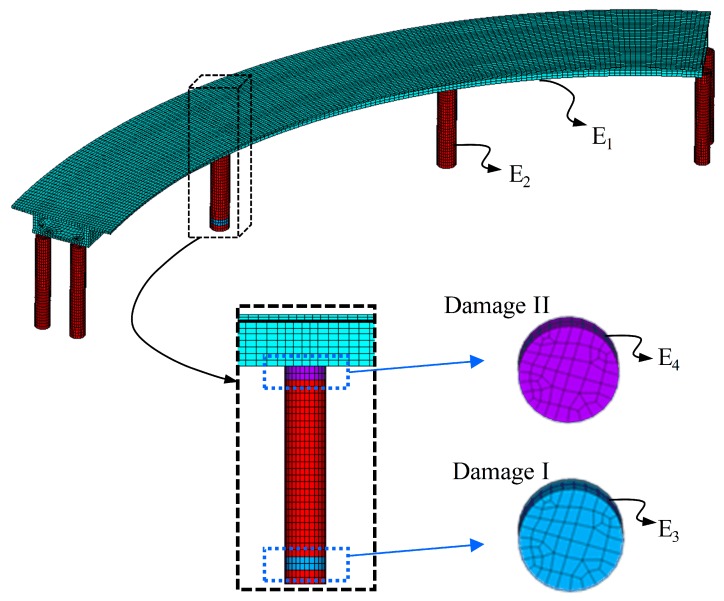
FE model of the investigated CCGB with zoomed-in Damage I and II ($E_{3}$, $E_{4}$ < $E_{2}$).

The geometry of the CCGB, including the plane, elevation, and cross sections, is illustrated in Figure 2. The finite element (FE) model of the bridge is built with 8-node 3D solid elements using the commercial software ANSYS, as presented in Figure 3. The model refers to the upper part of the ground with the boundary conditions of the fixed bottom end of the pier adopted. The material parameters for the FE model follow that the bridge deck is made of C50 concrete (elastic modulus $E_{1}=3.45\times 10^{4}\;\mathrm{MPa}$, Poisson’s ratio ν=0.2, and density $\rho =2500\;\mathrm{kg}/m^{3}$) and the other components are C40 concrete (elastic modulus $E_{2}=3.25\times 10^{4}\;\mathrm{MPa}$, Poisson’s ratio ν=0.2, and density $\rho =2500\;\mathrm{kg}/m^{3}$).

### 4.2. Seismic Damage Scenarios

Two typical damage instances, Damage I at the bottom of a pier and Damage II at a pier-girder connection, are introduced into the FE model to create damage cases. The damage is modeled by reducing the stiffness of relevant elements in damage areas, as shown in Figure 3. In fact, it is acknowledged that Damage I and II usually do not occur simultaneously for the same pier, as evidenced by many examples of bridge failure in practical engineering [50]. Thus, only a single damage scenario is considered in this study. Table 1 presents a set of sixteen damage scenarios, specified by individually reducing stiffness from 5% to 35% by steps of 5% to form Damage I and Damage II. For completeness, a fairly small reduction of stiffness, 0.01%, is considered for Damage I and Damage II simultaneously, with the purpose of modeling initial structural damage during manufacturing or concreting.

### 4.3. Seismic Excitation

**Figure 4:**
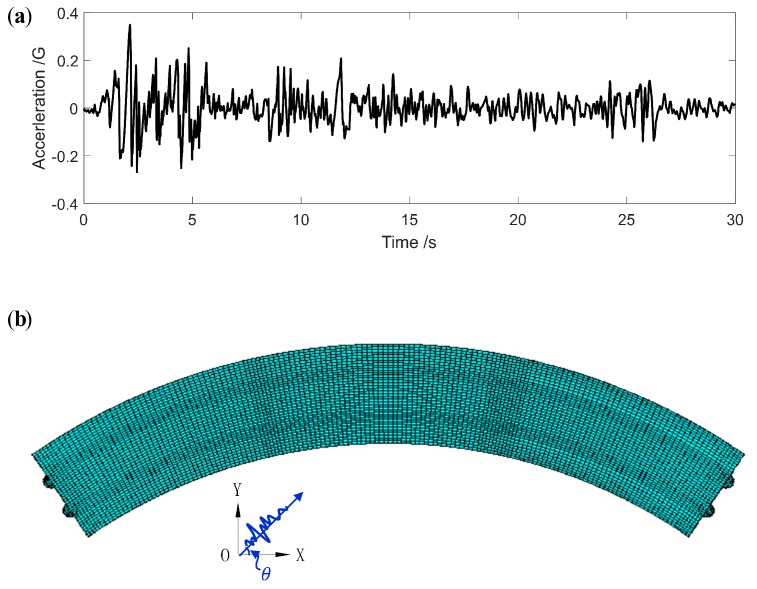
El Centro earthquake: (**a**) acceleration wave; (**b**) input angle.

The El Centro earthquake record from the El Centro Earthquake webpage [51] is adopted to excite the CCGB, which is considered as an inertial force. The acceleration wave is shown in Figure 4a. As an inertial force, the input angle of the wave has a significant impact on the maximum seismic response of the bridge. Empirically, the input angle, represented by θ in Figure 4b, is set as $\theta =45^{\circ }$.

### 4.4. Sensor Arrangement

**Figure 5:**
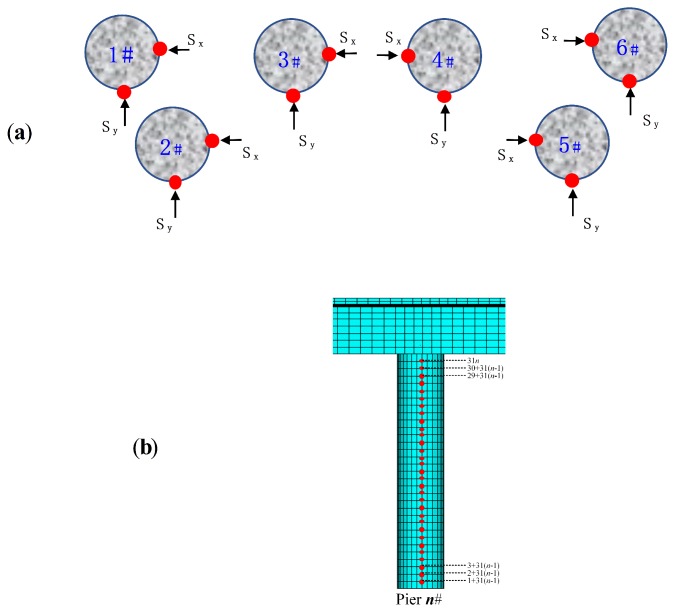
Sensor arrangement on CCGB piers: (**a**) pier number and sensor position; (**b**) vertical distribution of sensors.

The sensor arrangement is illustrated in Figure 5. Both *x*- and *y*-direction displacements of each pier are recorded by specific sensors, marked as $S_{x}$ and $S_{y}$ in Figure 5a. These sensors are numbered from 1+31(n−1) to 31n from bottom to top of piers, where *n* represents the pier ID in Figure 5a and 31 is the number of sensors on each pier, as illustrated in detail in Figure 5b. Herein, damage is introduced into pier 3#, measured by sensors 64–66 for Damage I and sensors 92–93 for Damage II.

**Figure 6:**
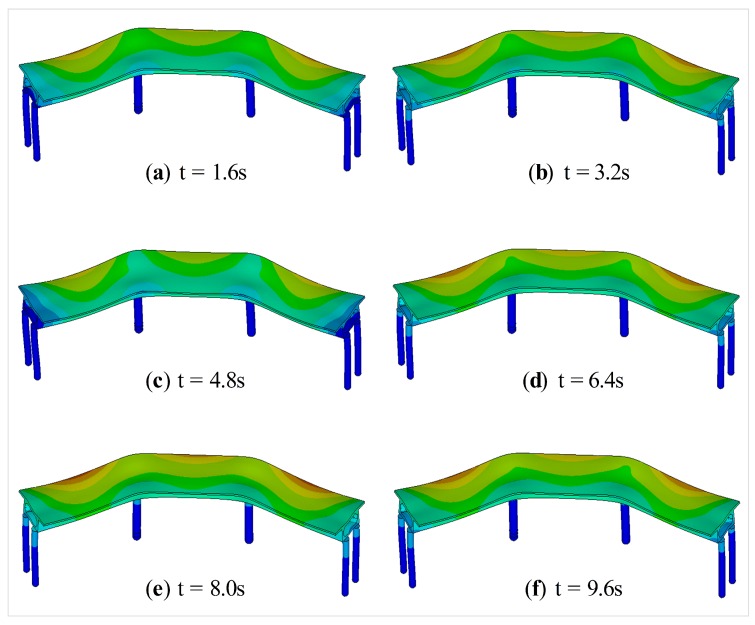
Displacement contour of the CCGB.

**Figure 7:**
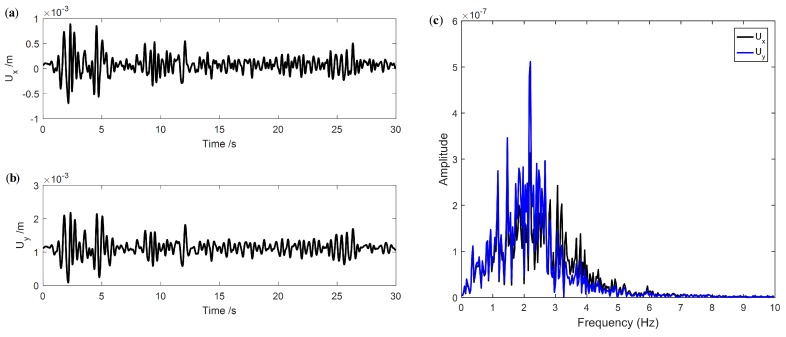
Displacement responses of the CCGB: (**a**,**b**) $U_{x}$ and $U_{y}$ in time domain; (**c**) $U_{x}$ and $U_{y}$ in frequency domain.

Figure 6 shows the displacement contour of the CCGB subjected to El Centro seismic excitation. The global trends of the displacement contours at different times are similar. The displacement responses at the top of pier 3# are presented in Figure 7. Figure 7a,b are the displacement responses in the *x*- and *y*-directions, represented by $U_{x}$ and $U_{y}$, manifesting that the overall trends are basically similar in the time domain, but with evident differences in magnitude. Figure 7c shows the $U_{x}$ and $U_{y}$ in the frequency domain. The dominant frequency of both $U_{x}$ and $U_{y}$ is 2.2 Hz according to Figure 7c.

## 5. Identification of Damage in CCGB

The WPSE-based damage identification method is utilized to identify Damage I and II in the CCGB described in Section 4, following the procedures presented in Figure 1.

### 5.1. Effective Seismic Responses

Structural damage identification is a typical inverse problem, with structural dynamic response as the object of analysis. There are many kinds of measurement data in practical engineering, such as acceleration, velocity and displacement. It is important to select an appropriate response type that is effective and sensitive to damage. In this study, $U_{x}$ and $U_{y}$, are obtained by applying the sensor arrangement as shown in Figure 5. When subjected to El Centro seismic excitation, $U_{x}$ and $U_{y}$ of pier 3# for Damage I are shown in Figure 7. It is clear that there is no significant difference between $U_{x}$ and $U_{y}$ in either time or frequency domains.

**Figure 8:**
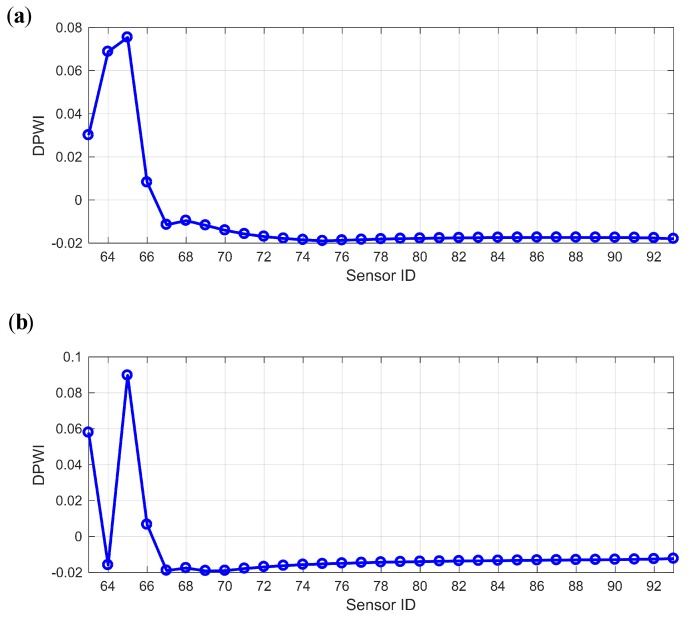
Comparison of DPWIs individually from $U_{x}$ and $U_{y}$: (**a**) $U_{x}$; (**b**) $U_{y}$.

The damage index DPWI, defined by Equation (15), is calculated using $U_{x}$ and $U_{y}$, with the results presented in Figure 8. As shown in both Figure 8a,b, the DPWI values are relatively large near the measurement points 64–66, indicating that damage may occur near this area. However, the DPWI curve in Figure 8a is smoother than that in Figure 8b, which fluctuates more due to the boundary effect. Therefore, $U_{x}$ is more suitable for damage identification and localization, and is adopted in the subsequent analysis in this study.

### 5.2. Optimal Wavelet Packet Parameters

The wavelet basis function and the decomposition scale are the most common wavelet parameters, both having important influences on the wavelet analysis results. The essence of wavelet analysis is to project the signal to a wavelet basis function, and the wavelet coefficients obtained characterize the dynamic features of the original signal. If the wavelet basis function is not properly selected, the unsuitable wavelet coefficients obtained reduce the accuracy of wavelet analysis. Simultaneously, the larger the decomposition scale, the higher the time frequency resolution of the signal, contributing to higher accuracy of the wavelet analysis. Nevertheless, this maneuver also increases the amount of calculation, resulting in information redundancy and a decrease in wavelet analysis efficiency. Therefore, the choice of optimal wavelet parameters is constantly an important topic in the field of wavelet analysis [52].

Currently, no uniform standard exists to determine optimum wavelet parameters. The Shannon entropy based cost function method is an effective method for determining the wavelet basis function. In this calculation, a Shannon entropy based cost function [53] is defined as

(16) $$M\left(s\right)=-\sum_{k=0}^{2^{j}-1}p_{k}log_{10}p_{k},$$

where $p_{k}=E_{j}^{k}\left(s\right)/\sum_{k=0}^{2^{j}-1}E_{j}^{k}\left(s\right)$ is the energy probability distribution of the wavelet coefficients, and $E_{j}^{k}\left(s\right)$ is the energy of the $k^{th}$ packet node at the $j^{th}$ decomposition level of signal *s*. For a given signal, the wavelet basis function corresponding to the lowest cost function value M is regarded as the optimal wavelet base. The decomposition scale can be considered comprehensively based on the cost function value and the calculation efficiency.

**Figure 9:**
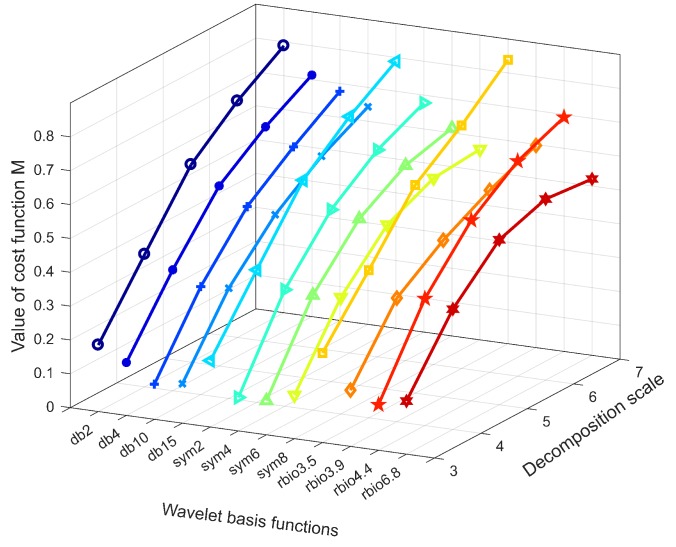
Value curves of the cost function M vs wavelet basis functions and decomposition scales.

In this study, twelve candidate wavelet basis functions, identified as db2, db4, db10, db15, sym2, sym4, sym6, sym8, rbio3.5, rbio3.9, rbio4.4, and rbio6.8, and five candidate optimal decomposition scales ranging from 3 to 7 are considered. The displacement response of the CCGB excited by El Centro seismic wave is taken as the analysis object. The values of cost function M are calculated using Equation (16), with the results presented in Figure 9. In accordance with Figure 9, rbio6.8 was determined to be the most appropriate basis for this case study because its M values of are relatively small within the global trend. There are no specific approaches for selecting the optimal decomposition scale. With synthetic consideration of the efficiency and accuracy of wavelet analysis, together with the ’trial-and-error’ method, level 6 was selected as the optimal decomposition scale in this study. Thus rbio6.8 and level 6 are used in the following analysis.

### 5.3. Effectiveness

**Figure 10:**
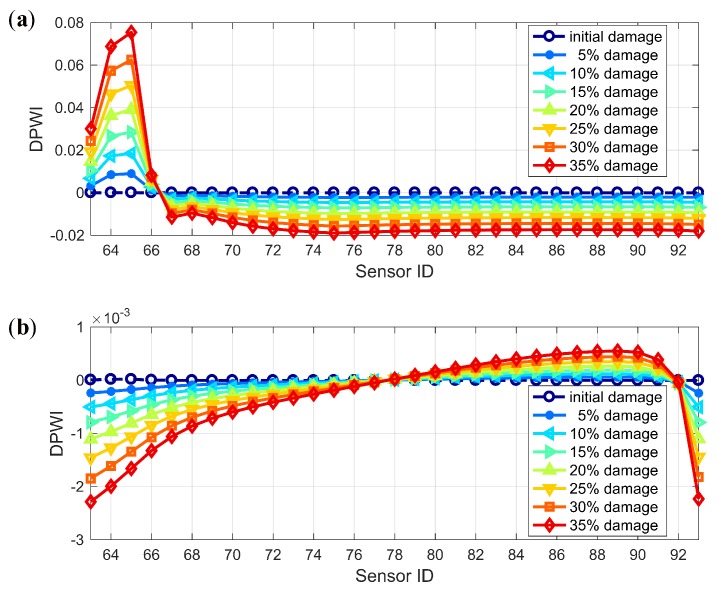
Damage identification results using DPWI: (**a**) Damage I; (**b**) Damage II.

As discussed in Section 3, the WPSE-based damage index DPWI can be used to characterize structural status, with the negative sign implying a healthy status and the positive sign implying an unhealthy status. Figure 10 shows the identification results for different damage levels using the DPWI. For the identification of Damage I as presented in Figure 10a, the DPWI only has an extreme value near the damage location, consistent with the damage criterion of DPWI > 0. However, for identification of Damage II as presented in Figure 10b, there is a wide range of positive DPWI near the damage location, and values of the DPWI become negative with a significant decline within the damage area. Therefore, the DPWI has certain damage recognition and localization capability for Damage I, but seems inappropriate for Damage II.

To make it applicable for identifying Damage II, the DPWI is modified following the curve feature presented in Figure 10b. According to the DPWI curve, significant decline occurs within the damage area only, and the curve integrity is well maintained in the area without damage. Thus, a modified index is constructed by adopting the second-order position derivative of DPWI:

(17) $$SDPWI_{i}=DPWI_{i+2}-2DPWI_{i+1}+DPWI_{i},$$

where *i* represents the position or number of the measuring point. The index SDPWI has the characteristics of removing the overall trend, highlighting the magnitude and maintaining the position information of any abrupt change.

**Figure 11:**
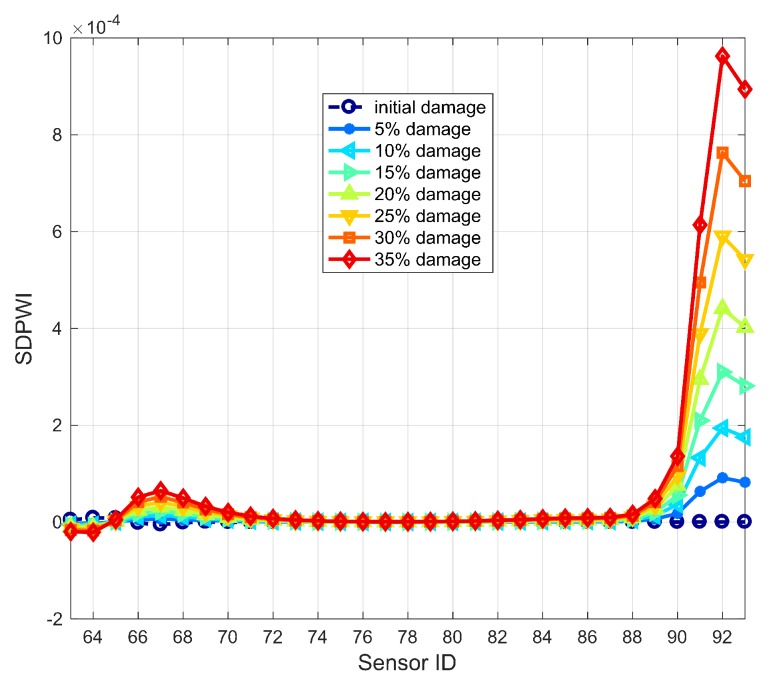
Damage identification results using SDPWI for Damage II.

Identification results of Damage II using the SDPWI are shown in Figure 11. The overall trend in Figure 10b has been successfully removed, and SDPWI has a maximum value within the damage area only. Therefore, the SDPWI is capable of identifying Damage II and has a high accuracy in localization of damage.

It must be noted that, although the SDPWI has advantages in identifying Damage II, it has the disadvantage of low noise immunity. This is because of performing the second order derivation, with the additional introduction of computational noise amplifying the noise influence. The noise immunity of the DPWI and SDPWI is discussed in detail in the next section.

On the basis of the DPWI and SDPWI, an early warning of damage can be triggered when the value of DPWI or SDPWI reaches the threshold of damage. The thresholds for the DPWI or SDPWI are set up independently. Here, the thresholds are taken as 0.8 DPWI, $6.2\times 10^{-3}$, and 0.8 SDPWI, $7.343\times 10^{-5}$, to warn of 5% seismic damage for Damage I at the bottom of piers and Damage II at pier-girder connections, respectively.

**Figure 12:**
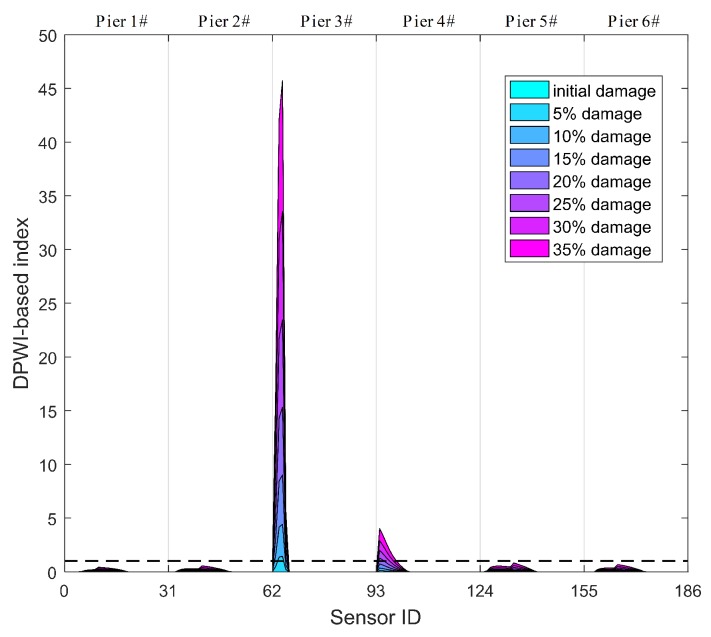
Identification results of Damage I for all six piers using warning values.

**Figure 13:**
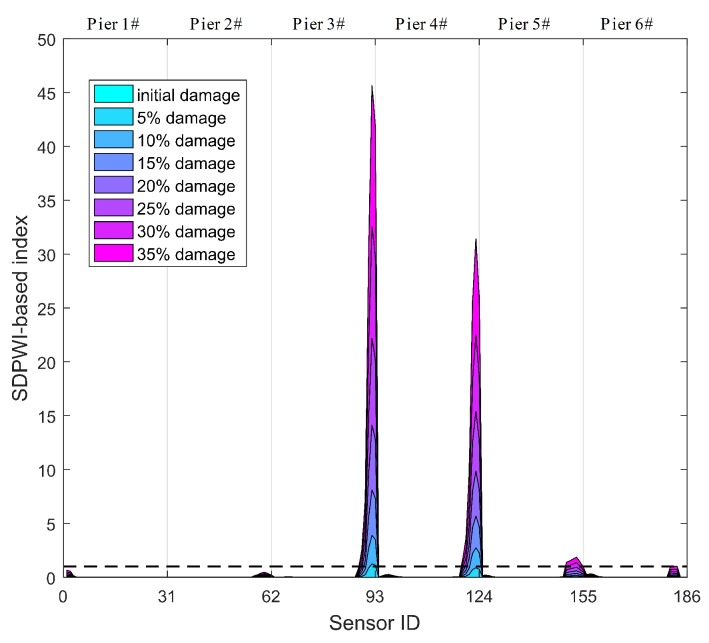
Identification results of Damage II for all six piers using warning values.

Specifically, those thresholds of the DPWI and SDPWI for damage warning are applied to seismic responses from six piers to identify damage at different piers. The identification results for Damage I and II are presented in Figure 12 and Figure 13, respectively, in which the normalized threshold level is marked with a black dotted line. In Figure 12, only the DPWI at the fixed end of pier 3# exceeds the threshold warning value, distinctly indicating the location of seismic damage. In Figure 13, the SDPWI exceeds the warning values not only in pier 3# but also in pier 4#, implying that pier 4# might also be damaged, which is inconsistent with the known seismic damage situation. This effect can be attributed to the symmetry of pier 3# and 4# along the curved structure, as illustrated in Figure 5.

## 6. Discussions

### 6.1. Comparison with Wavelet Packet Energy-Based Method

For a performance illustration, the proposed DPWI is compared to the wavelet packet energy (WPE) method [54] involving the damage index of WPE ($DI_{WPE}$):

(18) $$DI_{WPE}=\sqrt{\frac{\sum_{i=1}^{2^{N}}{\left(e_{i}^{d}-e_{i}^{h}\right)}^{2}}{\sum_{i=1}^{2^{N}}{\left(e_{i}^{h}\right)}^{2}}},$$

where $e_{i}^{h}$ and $e_{i}^{d}$ denote the energy level corresponding to healthy and damaged status, respectively.

**Figure 14:**
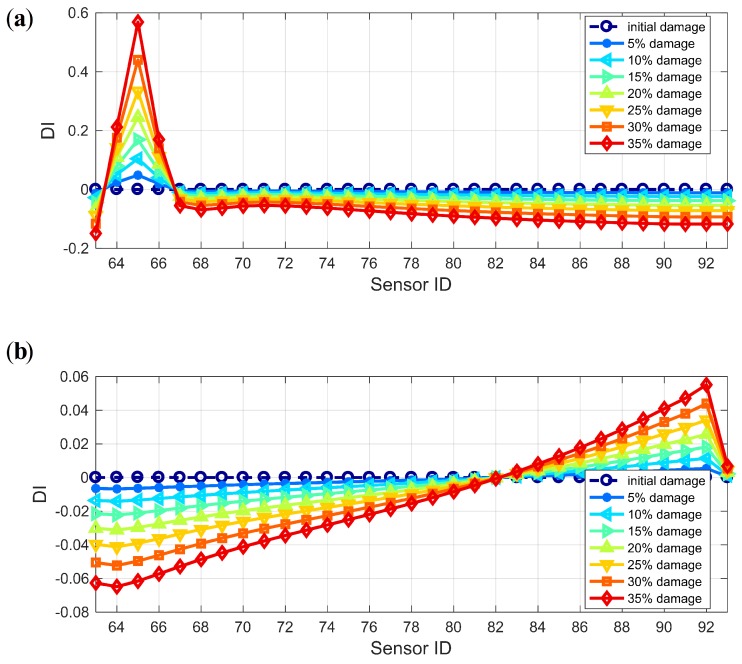
Damage identification results of the CCGB using WPE-based index DI: (**a**) Damage I; (**b**) Damage II.

Figure 14 presents the damage identification results using the $DI_{WPE}$ index. It is clear that Damage I is recognized correctly in Figure 14a, like the results shown in Figure 10a based on the DPWI index. However, following the damage criteria of $DI_{WPE}$>0, Damage II is not characterized accurately by the energy-based index $DI_{WPE}$, as shown in Figure 14b, which can be identified exactly with the SDPWI index as presented in Figure 11.

**Figure 15:**
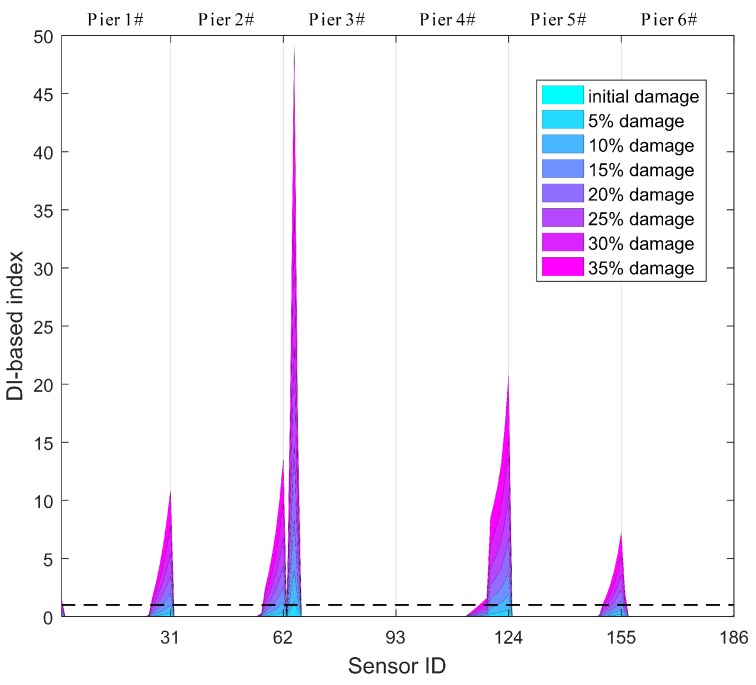
Identification results for Damage I using WPE-based method for all six piers.

**Figure 16:**
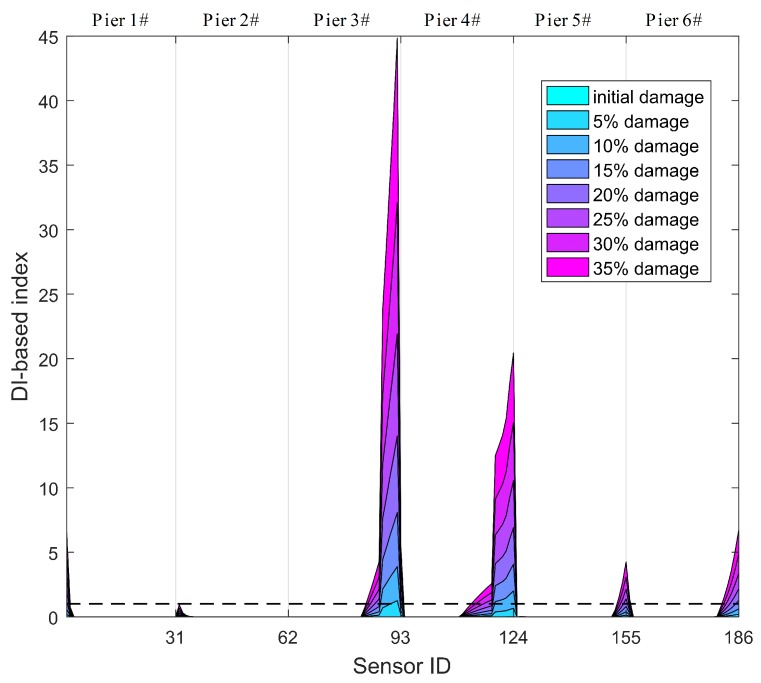
Identification results for Damage II using WPE-based method for all six piers.

The identification results for all piers using $DI_{WPE}$ for Damage I and II are shown in Figure 15 and Figure 16, respectively. It is clear that the WPE-based method is not as effective as the method based on WPSE, because redundant peaks occur in areas without damage or on healthy piers. Moreover, the boundary condition effect seems to be more obvious in the WPE-based method, in which the values of $DI_{WPE}$ near the boundaries are much greater than those in other areas. These findings provide evidence that the WPSE-based method provides a better result for evaluation of the condition of CCGBs.

### 6.2. Effect of Seismic Excitation

**Figure 17:**
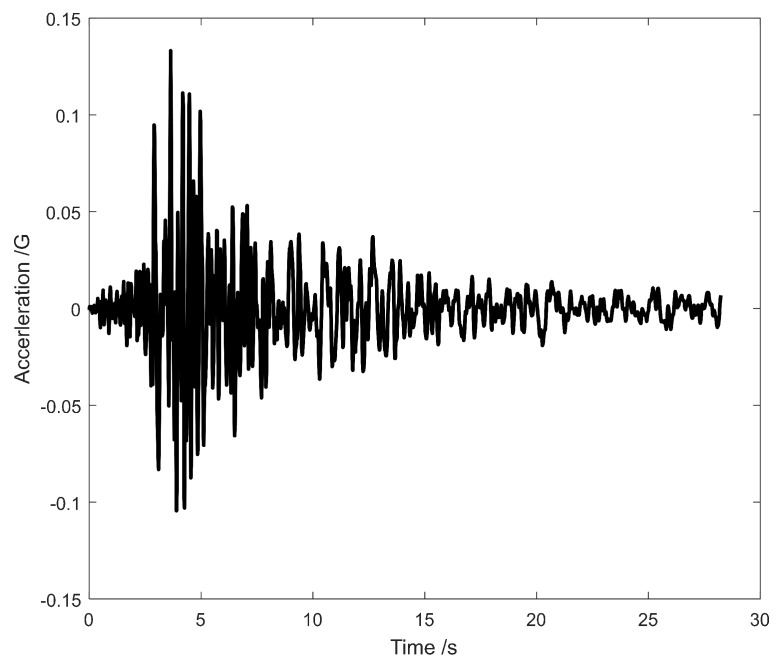
Acceleration history of Whittier Narrows earthquake.

**Figure 18:**
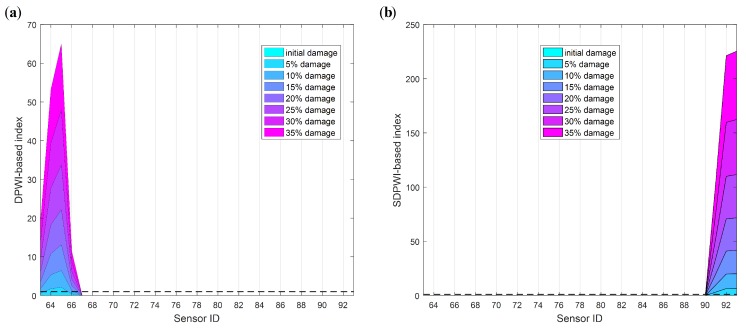
Damage identification results of the CCGB under Whittier Narrows earthquake: (**a**) Damage I, (**b**) Damage II.

The effect of seismic excitation is investigated to confirm the adaptability of the proposed method. The strong Whittier Narrows earthquake (Figure 17) is adopted as another seismic excitation for the FE model of the CCGB. Given the employment of the WPSE-based method, the identification results for Damages I and II in pier 3# are presented in Figure 18. It is clear that values of both DPWI and SDPWI designate damage correctly. This finding indicates that seismic excitations have no significant influence on the results of seismic damage identification.

### 6.3. Robustness against Noise

In practical applications, measured dynamic responses are inevitably polluted by noise. In the assessment of any damage identification method, effective identification of damage under noisy conditions is essential. Here, structural damage identification is undertaken using displacement responses with Gaussian white noise, and the robustness against noise of the WPSE-based damage detection method is discussed.

**Figure 19:**
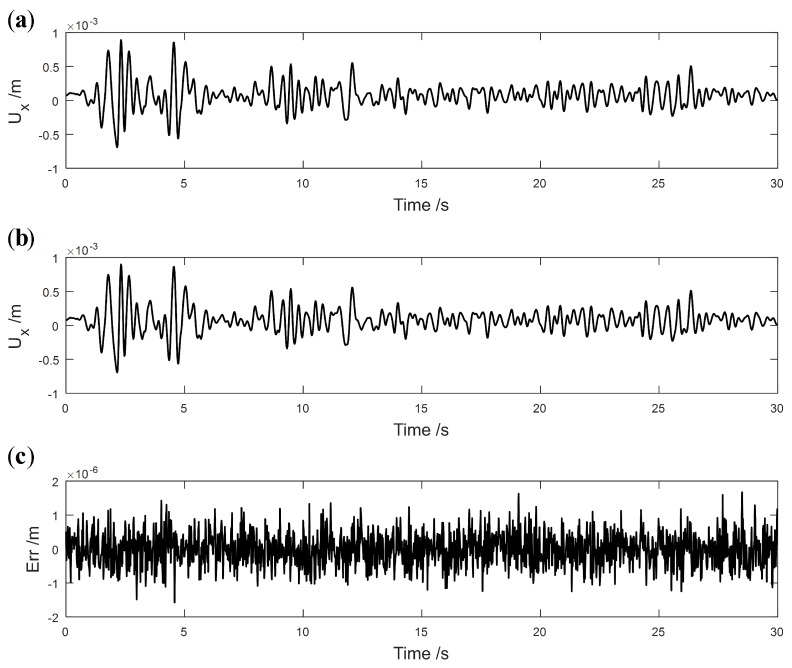
Comparison of noiseless and noisy signals of $U_{x}$ with *SNR* = 60 dB: (**a**) noiseless data; (**b**) noisy data; (**c**) differences between noiseless and noisy data.

**Figure 20:**
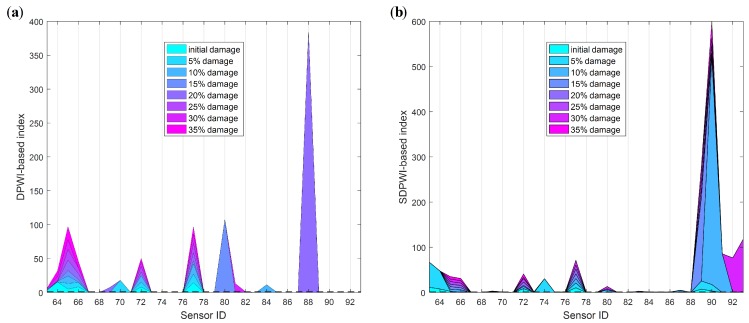
Damage identification results of the CCGB using noisy responses: (**a**) Damage I; (**b**) Damage II.

To label the noise level, a signal-to-noise ratio (SNR) definition is presented as

(19) $$SNR=20log_{10}\left(\frac{A_{Signal}}{A_{Noise}}\right),$$

where $A_{Signal}$ and $A_{Noise}$ denote the root-mean-square (RMS) magnitude of the vibration signal and added noise, respectively. Figure 19 shows the noiseless and noisy signals of $U_{x}$ with *SNR* = 60 dB. Figure 20 presents the identification results with the application of noisy responses for Damages I and II. As illustrated in Figure 20, the effectiveness of the structural damage detection method is diminished because redundant peaks appear in areas with no damage.

SVD is one of the most effective denoising tools. It can basically eliminate random noise and retain most of the useful information by choosing appropriate orders of singular values. This adaptive anti-noise technique can be used to enhance the robustness of WPSE-based indices because the WPSE inherits the characteristics of SVD, as discussed in Section 3.

**Figure 21:**
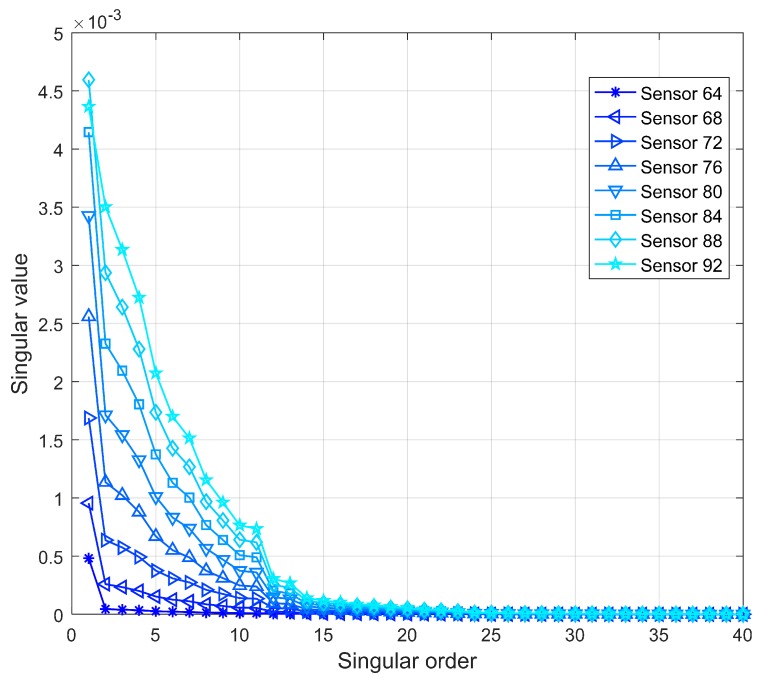
Wavelet packet singular values of noisy $U_{x}$ with SNR=60 dB.

**Figure 22:**
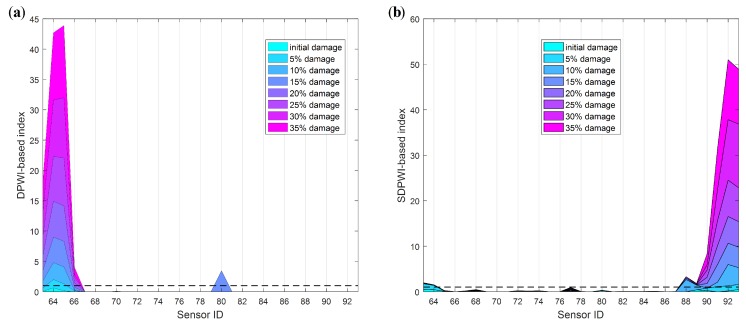
Damage identification results using first 5 singular values: (**a**) Damage I; (**b**) Damage II.

Wavelet packet singular values of $U_{x}$ with *SNR* = 60 dB are shown in Figure 21. Singular values of the order greater than 15 tend to be zero, and the predominant singular values are concentrated within orders 1 to 10. In this study, singular values of the fifth order are adopted in calculating the WPSE; that is, *q* equals 5 rather than the total number of singular values in Equations (11) and (12) when considering the adaptive anti-noise technique. The damage identification results with the SVD denoising technique for Damages I and II are presented in Figure 22. Compared with the results in Figure 20, the redundant peaks are eliminated and the damage location can be recognized correctly. Thus, the robustness of the damage indices DPWI and SDPWI against noise can be significantly enhanced by use of the adaptive anti-noise technique without additional noise reduction techniques.

Monte Carlo simulations are performed at different SNR levels to determine the maximum noise immunity and damage identification accuracy of the proposed method in noisy conditions. Two indices, the missing report rate (MRR) and the accuracy of damage warning (ADW), are established to quantitatively represent the accuracy of damage detection. They are calculated in accordance with the following formulas:

(20) $$MRR=\frac{\sum_{i=1}^{N}n_{i}}{N}\times 100\%,$$

(21) $$ADW=\frac{\sum_{i=1}^{N}c_{i}/m_{i}}{M}\times 100\%,$$

where *N* is the total number of Monte Carlo simulations; *M* is the total number of occurrences of warning during *N* simulations; $m_{i}$ is the number of early warnings reported in the $i^{th}$ simulation; $n_{i}$ is the number of missed damage reports in the $i^{th}$ simulation; $c_{i}$ is the number of correct warnings in the $i^{th}$ simulation.

In Equations (20) and (21), MRR refers to the probability that damage has occurred but is not detected, and ADW refers to the probability of correctly identifying damage location when an early warning occurs. At different noise levels, a stronger robustness damage identification method corresponds to a lower MRR and a higher ADW.

In this study, N=1000 simulations are performed with SNR ranging from 40 dB to 90 dB for Damages I and II. The results are listed in Table 2. From Table 2, MRR is generally less than 5%, indicating the damage is to a great extent detected when it occurs. However, for damage scenario 3, the failure rate MRR is increased to 15% by *SNR* = 50 dB, implying that the identification of damage below 5% will result in a greater error. In terms of ADW, the accuracy of early warning of damage is generally greater than 90% for *SNR* = 50 dB, but the accuracy of early warning of damage decreases quickly to less than 80% for *SNR* = 40 dB. Therefore, the damage indices DPWI and SDPWI based on WPSE proposed in this paper are suitable for *SNR*≥ 50 dB.

To simplify the application of MRR and ADW, a joint-index MA is defined as

(22) $$MA=\left(1-MRR\right)\cdot ADW.$$

If we select MA = 0.9 as the accuracy criterion, the results presented in Table 3 demonstrate that the proposed method can identify damage in the presence of SNR ≥ 50 dB with high precision.

## 7. Conclusions

In this study, two WPSE-based evaluation indices were proposed to identify seismic damage in CCGBs, by taking synergistic advantage of the wavelet packet transform, singular value decomposition, and information entropy. The effectiveness of the proposed approach was numerically verified by a finite element model of a real CCGB subjected to El Centro seismic excitation. Numerical results showed that the two WPSE-based indices, DPWI and SDPWI, are capable of identifying the existence of damage and can locate damage at the bottom of piers and at pier-girder connections, respectively. Moreover, it was demonstrated that the robustness of the proposed indices against noise were enhanced by application of the adaptive anti-noise technique, specifically by choosing the first 5 singular values during WPSE analysis. In addition, the Monte Carlo simulation results suggested that the WPSE-based approach can effectively detect seismic damage in noisy conditions with *SNR*≥ 50 dB. The benefit of the WPSE-based method was further clarified by comparisons with the WPE-based method. The comparison results showed that the WPSE-based method recognized the location of damage with higher accuracy. Thus, the proposed WPSE-based approach is reliable and applicable to seismic damage identification in CCGBs. This WPSE-based approach holds significant promise as a support to advanced distribution sensing techniques, especially to the distributed optical fiber sensors used in SHM of critical civil infrastructures.

## Figures and Tables

**Table 1:** Seismic damage scenarios for the CCGB.

No.	Damage Type		Damage Severity								
	I	II	0%	0.01%	5%	10%	15%	20%	25%	30%	35%
1			☆								
2	☆	☆		☆							
3		☆			☆						
4		☆				☆					
5		☆					☆				
6		☆						☆			
7		☆							☆		
8		☆								☆	
9		☆									☆
10	☆				☆						
11	☆					☆					
12	☆						☆				
13	☆							☆			
14	☆								☆		
15	☆									☆	
16	☆										☆

’☆’ represents the damage type and severity for each corresponding damage scenario.

**Table 2:** Monte Carlo simulation results for different noise levels.

Damage	*MRR* (%)						*ADW* (%)					
Scenario	*SNR* (dB)						*SNR* (dB)					
No.	40	50	60	70	80	90	40	50	60	70	80	90
3	32.40	15.00	4.80	1.60	0.30	0.20	92.75	98.00	99.26	99.59	99.90	100.00
4	9.80	3.70	1.20	0.40	0.10	0.00	94.90	98.23	99.49	99.60	99.80	100.00
5	6.10	1.80	1.10	0.40	0.00	0.00	95.21	99.39	99.80	99.80	100.00	100.00
6	3.90	1.10	0.70	0.00	0.10	0.00	95.84	99.29	99.50	100.00	100.00	100.00
7	2.10	0.90	0.30	0.00	0.00	0.00	96.53	99.39	99.70	99.80	100.00	100.00
8	1.40	0.50	0.30	0.00	0.00	0.00	96.75	99.30	99.60	99.90	100.00	100.00
9	1.50	0.40	0.10	0.00	0.00	0.00	96.95	98.90	99.50	99.90	100.00	100.00
10	2.90	1.40	0.60	0.20	0.00	0.00	65.81	87.93	95.88	98.40	99.60	99.80
11	0.00	0.00	0.00	0.00	0.00	0.00	74.50	90.70	96.70	98.90	99.90	99.90
12	0.00	0.00	0.00	0.00	0.00	0.00	80.80	93.80	98.10	99.10	99.90	100.00
13	0.00	0.00	0.00	0.00	0.00	0.00	82.10	93.80	98.10	99.40	100.00	99.90
14	0.00	0.00	0.00	0.00	0.00	0.00	82.90	94.10	97.70	99.20	99.90	100.00
15	0.00	0.00	0.00	0.00	0.00	0.00	85.80	94.80	98.00	99.40	99.90	100.00
16	0.00	0.00	0.00	0.00	0.00	0.00	86.10	94.70	98.10	99.40	100.00	100.00

**Table 3:** MA values from Monte Carlo simulations for different noise levels.

Damage	MA					
Scenario	*SNR* (dB)					
No.	40	50	60	70	80	90
3	0.627	0.833	0.945	0.980	0.996	0.998
4	0.856	0.946	0.983	0.992	0.997	1.000
5	0.894	0.976	0.987	0.994	1.000	1.000
6	0.921	0.982	0.988	1.000	0.999	1.000
7	0.945	0.985	0.994	0.998	1.000	1.000
8	0.954	0.988	0.993	0.999	1.000	1.000
9	0.955	0.985	0.994	0.999	1.000	1.000
10	0.639	0.867	0.953	0.982	0.996	0.998
11	0.745	0.907	0.967	0.989	0.999	0.999
12	0.808	0.938	0.981	0.991	0.999	1.000
13	0.821	0.938	0.981	0.994	1.000	0.999
14	0.829	0.941	0.977	0.992	0.999	1.000
15	0.858	0.948	0.980	0.994	0.999	1.000
16	0.861	0.947	0.981	0.994	1.000	1.000
